# The effect of immunohistochemically detected p53 accumulation in prognosis of breast cancer; A retrospective survey of outcome

**DOI:** 10.1371/journal.pone.0182444

**Published:** 2017-08-03

**Authors:** Sanambar Sadighi, Mohammad Zokaasadi, Amir Kasaeian, Somaye Maghsudi, Issa Jahanzad, Hosein Kamranzadeh Fumani

**Affiliations:** 1 Department of Medical Oncology, Cancer Research Center, Cancer Institute of Iran, Tehran University of Medical Sciences, Tehran, Iran; 2 Hematology, Oncology and Stem Cell Transplantation Research Center; Tehran University of Medical Sciences, Tehran, Iran; 3 Department of Pathology, Cancer Research Center, Cancer Institute of Iran, Tehran University of Medical Sciences, Tehran, Iran; University of North Carolina at Chapel Hill School of Medicine, UNITED STATES

## Abstract

**Background:**

P53; a tumor suppressor gene has known to have a role in a group of human cancers. Its role in breast cancer; one of the most prevalent malignancies worldwide, is still controversial. The current study is designed to evaluate the prognostic role of p53 mutation in breast cancer.

**Methods:**

one hundred and eighty five breast cancer patients were studied in this retrospective study. P53 mutation was detected by accumulation of p53 protein in the patients’ pathology samples. Immunohistochemistry (IHC) was used to detect the protein. The effect of p53 on the final outcome was assessed using Kaplan-Meier estimate of survival and compared by log-rank test. Prognostic effects analyzed by cox proportional hazard models.

**Results:**

while the stage of the disease at presentation was not significantly different between p53 positive and negative patients, those with p53 mutation had a significantly poorer outcome in terms of overall and disease-free survival rates (OS and DFS). In a multivariate analysis hazard ratio of p53 mutation was about 5 and 3.8 for OS and DFS respectively. They also had a higher cumulative incidence of relapse.

**Conclusion:**

It seems that p53 mutation is an independent prognostic factor for breast cancer. Although larger prospective studies are needed to clarify the importance of such a conclusion.

## Introduction

Breast cancer is the most common type of cancer diagnosed among women in the USA and globally [1] and the second cause of cancer-related mortality in women in the united stated of America in the current decade [2] [3]. It is also the cardinal cause of disability adjusted life years (DALY) based on worldwide studies [4]. Epidemiological studies have shown that breast cancer is the most common cancer type among Iranian women with an age standardized rate (ASR) of 17.1 to 24 per 100,000 [5] [6]. Moreover breast cancer affects Iranian women about one decade earlier compared to developed countries [7]. This disease includes a number of biologically diverse subtypes [8]. This very diversity explains the different therapeutic approaches and prognosis of the disease. There are different biomarkers postulated to have a role in the clinical and therefore therapeutic aspects of the disease i.e. cathepsin-D, Ki-67 and p53. One of the most studied ones is p53; a tumor suppressor protein coded by a gene called Tp53[9]. This gene is placed on the short arm of chromosome 17[10]. However there is no agreement on the p53 prognostic importance in breast cancer yet. Most of previous experiences claimed the effect of carrying a mutated p53 protein on death and recurrence [11–13], while some others did not [14].

The current study is a retrospective cohort designed to assess the outcome of patients with early breast cancer focusing on p53 prognostic effect.

## Materials and methods

From March 2007 to July 2013, 187 newly diagnosed patients with breast cancer were included in the study. Exclusion criterion was male breast cancer. Values including patient age, menstruation status, surgery type, lymph node involvement, tumor size, stage, chemotherapy, radiation therapy and hormonal treatment were extracted from medical records. Receptors of estrogen (ER), progesterone (PR) and Human Epidermal growth factor (HER2/neu) had been assayed by immunohistochemistry (IHC) and the pertinent data were extracted from patients’ files. P53 assays were done using IHC as well (Code M3629, Dako, Germany). Validation of assay was performed through the following steps: the tests were done in 1/50 concentration, Ph = 9 and all steps were done and controlled based on the manufacturer’s instructions.

All samples are reviewed by a second pathologist and the diagnosis were confirmed. Grade of tumors was defined based on Elston-Ellis method [15]. In this grading system tubule formation, mitotic activity and nuclear pleomorphism were used in order to categorize differentiation of cells in three groups of well, moderately and poorly differentiated. Outcome categorized in one of the following: distant or metastatic recurrence, local recurrence, death and none. Informed consent was obtained from all patients in order to use their medical records as a source for medical research. The study was approved by ethical committee of Tehran University of Medical Sciences. For the sake of analysis pathologic grade divided into two categories: grade 3 in one group and grades 1 or 2 in another. After calculating a total overall survival rate, metastatic cases are dropped from analysis in order to run more detailed analysis considered disease-free survival (DFS) and cox proportional hazard models. All treatments and follow up visits took place in Cancer Institute of Iran, Tehran University of Medical Sciences, Tehran, Iran.

Data were analyzed retrospectively and Kaplan-Meier estimate were used to evaluate survival rates (both overall and disease-free). Median survival time was calculated by reverse Kaplan-Meier method. Cox proportional hazard models were used to evaluate the effect of prognostic factors on the outcome and each prognostic factor with a P-value of less than 0.2 was authenticated to commence a multivariate analysis. Cumulative incidences of relapse were calculated by Gray’s method and the groups were compared by the Gray test method in the competing risks setting [16]. In analyses P-value of less than 0.05 is considered statistically significant. All analyses were performed using Stata software version 11.2 And R version 3.3.2 for windows.

## Results

### Cohort characteristics

A total number of 185 patients included in the study. Mean age at diagnosis was 47.19±11.42 years. P53 was detected positive in 35.14% of cases (n = 65). Twenty patients had distant metastasis (stage 4) at the time of diagnosis (10.81%). Pathologic examination revealed grade 2 disease in 77.84% of the patients (n = 144). Sixteen patients (8.65%) underwent biopsy only and the remaining had surgery (mastectomy or breast-conserving). Basic characteristics were summarized in Table 1;

**Table 1:** Baseline characteristics of patients.

*covariate*	*classification*	*frequency*
Menopausal status	Pre-menopause	68.65%(n = 127)
	Menopause	31.35%(n = 58)
*Tumor T*	T1	22.16%(n = 41)
	T2	52.43%(n = 97)
	T3	14.59%(n = 27)
	T4	8.65%(n = 16)
	T_x_	2.16(n = 4)
*Lymph node involvement*	N0	34.59%(n = 64)
	N1	31.35%(n = 58)
	N2	16.76%(n = 31)
	N3	9.73%(n = 18)
	N_x_	7.57%(n = 14)
*Distant metastasis*	M0	89.19%(n = 165)
	M1	10.81%(n = 20)
*stage*	1	12.97%(n = 24)
	2	48.11%(n = 89)
	3	28.11%(n = 52)
	4	10.81%(n = 20)
*Pathologic grade*	1	2.70%(n = 5)
	2	77.84%(n = 144)
	3	19.46%(n = 36)
*Estrogen receptor*	Positive	66.49%(n = 123)
	Negative	33.51%(n = 62)
*Progesterone receptor*	Positive	61.62%(n = 114)
	Negative	38.38%(n = 71)
*HER2/neu*	Positive	23.24%(n = 43)
	Negative	76.76%(n = 142)
*Surgery type*	MRM*	76.22%(n = 141)
	BCS*	15.14%(n = 28)
	Biopsy only	8.65%(n = 16)
*Relapse*	No recurrence	71.89%(n = 133)
	Local recurrence	4.86%(n = 9)
	Distant recurrence	12.97%(n = 24)
	N/A**	10.27%(n = 19)
*Treatment*	Chemotherapy	97.39%(n = 182)
	Radiotherapy	68.11%(n = 126)
	Hormone therapy	64.32%(n = 119)
*P53*	Positive	35.14%(n = 65)
	Negative	64.86%(n = 120)

*MRM: modified radical mastectomy, BCS: breast conserving surgery.

**Patients with an advanced metastatic disease from the time of diagnosis.

There was no significant association between p53 status at the time of diagnosis with the following variables: stage of disease, menopausal status, progesterone receptor and HER2 (Chi-square, P-value = 0.68, 0.84, 0.11 and 0.30 respectively). Median follow up time was 47±1.35 months with a maximum of 75 months.

### Survival analysis

At the end of the follow up time 33 patients had a regional or distant relapse. There were also 31 cases of death. Kaplan-Meier estimate showed 5-year overall (OS) rate of 80.77% (95% CI: 73.25%-86.37%).

**Fig 1:**
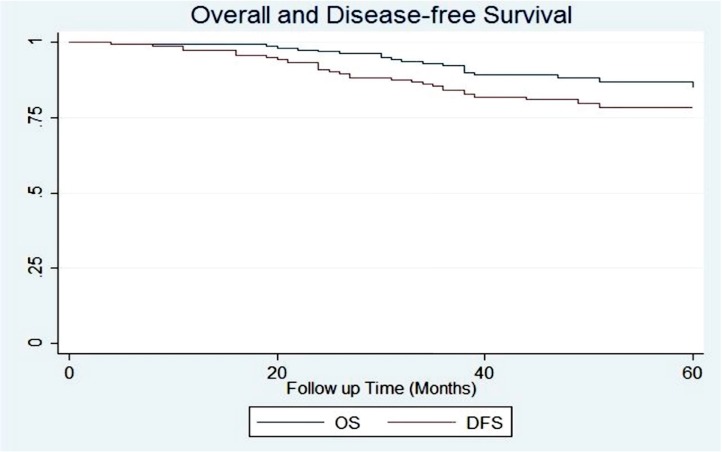
Overall and disease-free survival of non-metastatic patients.

After dropping metastatic cases from analysis a total number of 165 patients were remained. Five-year OS and DFS rates for the selected population were 86.68% (95% CI: 79.29%-91.57%) and 78.15% (95% CI: 69.92%-84.38%) respectively. Fig 1.

**Fig 2:**
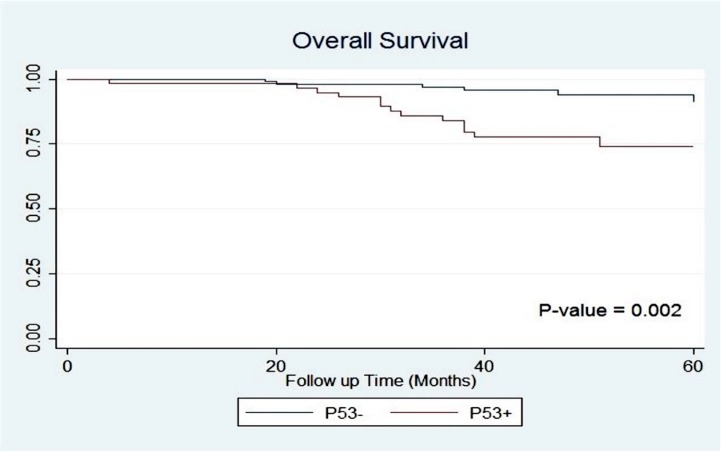
Overall survival of patients based on p53 accumulation.

**Fig 3:**
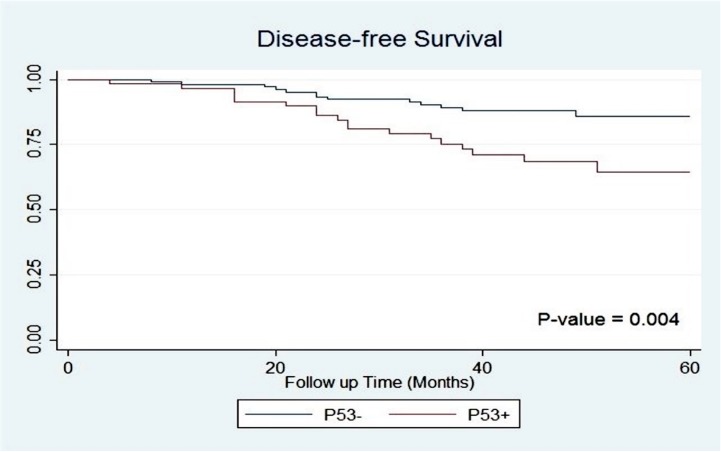
Disease-free survival of patients based on p53 accumulation.

Significant differences were observed between p53 positive and negative groups for OS and DFS (5-year OS: 73.87% vs. 94.03%, P = 0.002; 5-year DFS: 64.25% vs. 85.89%, P = 0.004); Figs 2 and 3.

Subgroup analysis did not show any significant differences for the effect of p53 on OS and DFS when stratified based on hormone receptor, HER2/neu or stage.

Additionally, significant differences was detected between overall survival of the patients by disease stage (P = 0.005), lymph node involvement (P = 0.007), estrogen receptor (P = 0.012) and pathologic grade (P = 0.004). In addition to these variables T category and progesterone receptor were perceived to be significant for DFS (P = 0.000 and 0.004 respectively). Results of survival analyses for different variables regarded to OS and DFS rates are summarized in Table 2;

**Table 2:** Variations in survival of patients in different subgroups.

Covariate	subgroups	5-year OS	P-value	5-year DFS	P-value
P53	Positive	73.87%	0.002	64.25%	0.004
	Negative	94.03%		85.89%	
T	T1	90.59%	0.098	81.39%	0.000
	T2	87.80%		83.36%	
	T3	82.08%		69.11%	
	T4	57.14%		25.71%	
Lymph node	N0	95.85%	0.007	90.86%	0.001
	N1	86.52%		86.52%	
	N2	77.91%		54.35%	
	N3	70.32%		51.85%	
Grade	1 or 2	89.20%	0.004	82.10%	0.013
	3	71.17%		56.54%	
Stage	Stage1	88.46%	0.005	82.45%	0.000
	Stage2	93.95%		92.11%	
	Stage3	73.07%		51.91%	
ER	Positive	91.07%	0.012	83.67%	0.013
	Negative	77.79%		67.23%	
PR	Positive	90.25%	0.064	85.37%	0.004
	Negative	81.25%		67.24%	
HER2/neu	Positive	80.28%	0.378	68.78%	0.23
	Negative	88.64%		80.85%	
Menopause	Pre-menopause	87.84%	0.94	77.17%	0.701
	Post-menopause	84.40%		78.37%	

### Univariate and multivariate analyses:

First the effect of different variables on outcome were assessed by means of univariate cox regression in order to select proper variables for multivariate analysis and build a more reliable model. Then multivariate analysis was used for variables with a P-value of 0.2 or less. Using a univariate analysis tumor size, disease stage, pathologic grade, p53 accumulation and hormone receptor status were selected to perform multivariate analysis. Finally adjusted for all other variables p53 mutation was found to be an independent factor predicting poor prognosis (hazard ratio for OS: 5.04 (95% CI: 1.71–14.94); P = 0.003 and for DFS: 3.81 (95% CI: 1.68–8.64); P = 0.001). Additionally stage 2 disease was found to be a prognosticator of superior disease free survival when compared to advanced stages (HR: 0.23 (95% CI: 0.06–0.90); P = 0.03). The results of univariate and multivariate analysis model are summarized in Table 3;

**Table 3:** Results of univariate and multivariate analyses.

Covariate			OS				DFS				
			Univariate		Multivariate		Univariate		Multivariate		
			HR (CI 95%)	P-value	HR (CI 95%)	P-value	HR (CI 95%)	P-value	HR (CI 95%)		P-value
**Age**			0.98(0.94–1.02)	0.35			1.00(0.97–1.03)	0.84			
**Size**			1.19(1.15–1.45)	0.07	1.00(0.78–1.28)	0.98	1.15(0.98–1.34)	0.09	0.94(0.77–1.15)		0.54
**Grade (3 over 1 or 2)**			3.66(1.44–9.29)	0.01	2.93(0.91–9.46)	0.07	2.57(1.18–5.58)	0.02	2.61(0.97–7.00)		0.06
**Stage**	2		0.73(0.14–3.74)	0.70	0.40(0.07–2.37)	0.32	0.39(0.11–1.38)	0.14	0.23(0.06–0.90)		0.03
	3		3.26(0.73–14.63)	0.12	2.22(0.36–13.63)	0.39	2.81(0.97–8.20)	0.06	2.11(0.56–8.00)		0.27
**ER or PR(Positive over Negative)**			0.33(0.13–0.82)	0.02	0.42(0.15–1.18)	0.10	0.42(0.21–0.85)	0.02	0.48(0.20–1.14)		0.10
**HER2/neu(Positive over Negative)**			1.54(0.58–4.06)	0.38			1.57(0.74–3.34)	0.24			
**P53**			4.22(1.60–11.11)	0.00	5.04(1.71–14.94)	0.003	2.74(1.34–5.60)	0.01	3.81(1.68–8.64)		0.001
**Menopause (menopause over pre-menopause)**			0.96(0.37–2.54)	0.94			1.15(0.55–2.40)	0.70			

### Relapse incidence

**Fig 4:**
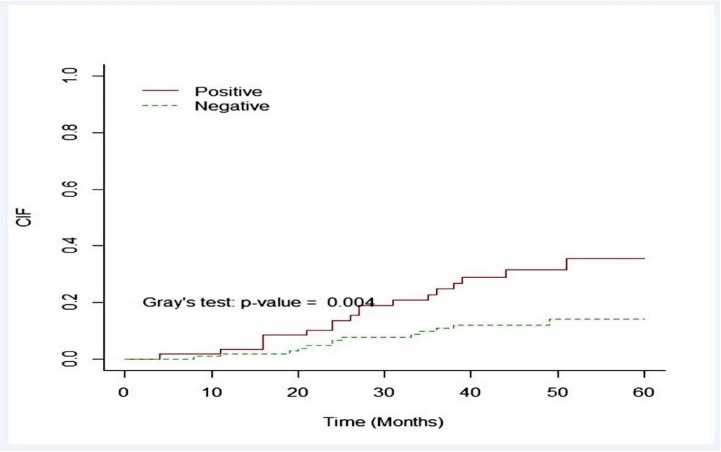
Cumulative incidences of relapse based on p53 status.

The cumulative 5-year incidence of relapse was 18.99% (95% Confidence interval: 15.00%-29.19%) which was significantly higher in p53 positive group (11.95% for p53 negative and 31.58% for p53 positive group; Gray’s test, P-value = 0.004; Fig 4 and Table 4)

**Table 4:** Cumulative relapse incidences.

Relapse incidence(%CI)	1-year	3-year	5-year
p53 positive	3.39(0.62–10.49)	24.71(14.29–36.61)	35.38(21.64–49.40)
p53 negative	1.89(0.36–6.05)	10.78(5.68–17.72)	14.00(7.61–22.30)

## Discussion

The primary aim of this study was to evaluate the frequency of p53 mutation and its effect on outcomes of patients with breast cancer. Our patients had a 35% p53 mutation which is in line with the literature, like 37% in study of Gasparini G et al [17]. Besides our results revealed a 5-year overall survival rate of 80.77%; which is an acceptable rate. We are also able to deduce that the pathologic grade is one of the independent prognostic factors for female breast cancer (although with a borderline P-value of 0.07 for OS and 0.06 for DFS). This finding is strongly supported by evidences published in the literature like a systematic review by Mirza AN et al on long term studies [18].

Moreover we detected the independent prognostic impact of IHC detected p53 mutation on the OS and DFS. Some other previous reports have reached to similar results. The report of Silvestrini et al on 256 node negative breast cancer patients with a long term follow up time of 72 months suggested that the p53 overexpression detected by IHC could be used along with other conventional prognostic factors [13]. The study of Blaszyk H et al on 90 Caucasian patients for a median follow up of 5 years revealed that the most prognostic factor in primary invasive breast cancer is p53 mutation [11]. Long-term study of Falette N et al on 113 patients for 105 months also confirmed the prognostic value of p53 mutation on both OS and DFS rates [19]. Moreover a prominent experience in node negative breast cancer patients in South Korea have shown that IHC detected p53 accumulation is of prognostic importance after adjusting for other possible factors [12]. On the other hand there are other opposing reports in the literature[20]. The study of Korkolis DP et al on 125 patients during a mean follow up time of 62 months showed that p53 accumulation is not related to the outcome [14]. These differences maybe to some degrees ascribable to different techniques for detection of p53 mutation. For instance one study performed on 188 high risk breast cancer patients revealed paradoxical results with two different models of classification; once the weakly positive results considered positive and then negative [21]. The latter one was not able to demonstrate any prognostic effect for p53 mutation.

Another method frequently and recently used for p53 mutation detection is based on genetic tests. A number of more recent studies have shown that only Tp53 gene mutations are accompanied with a worse outcome and other alterations like p53 mRNA expression are of less significance[22]. Thus it can be deduced that maybe the use of DNA sequencing methods for detecting mutations in Tp53 are more accurate than the IHC, the idea which is supported by the study of Sjogren S et al [23]. However, the IHC method is cost efficient and it worked suitably in our cohort and some other previous studies as well, so it can be considered as a technique that costs less and does not need highly experienced technicians.

Moreover we found out that p53 status is not related to more invasive disease at the onset; a judgment which is supported by evidences in the literature. Studies of Mylonas I et al and Liu C et al also showed that the expression of p53 in invasive mammary ductal carcinoma does not significantly differ from the one in ductal carcinoma in situ [24, 25]. Additionally an observational study from Iran revealed that the p53 status is not significantly related to lymph node involvement [26]. Therefore it can be offered that the prognostic value of p53 mutation should be interpreted per se and distinctively from clinicopathological features.

According to biologically diverse types of breast cancers and also the potential role of a large group of other risk factors it seems that more detailed studies are needed to assess this prevalent disease and its outcome in the future.

## References

[1] Torre LA, Siegel RL, Ward EM, Jemal A. Global Cancer Incidence and Mortality Rates and Trends—An Update. Cancer epidemiology, biomarkers & prevention: a publication of the American Association for Cancer Research, cosponsored by the American Society of Preventive Oncology. 2016;25(1):16–27. doi: 10.1158/1055-9965.EPI-15-0578 . 26667886 10.1158/1055-9965.EPI-15-0578

[2] Jemal A, Siegel R, Xu J, Ward E. Cancer statistics, 2010. CA: a cancer journal for clinicians. 2010;60(5):277–300. doi: 10.3322/caac.20073 . 20610543 10.3322/caac.20073

[3] Siegel RL, Miller KD, Jemal A. Cancer statistics, 2016. CA: a cancer journal for clinicians. 2016;66(1):7–30. doi: 10.3322/caac.21332 . 26742998 10.3322/caac.21332

[4] Global Burden of Disease Cancer C, Fitzmaurice C, Allen C, Barber RM, Barregard L, Bhutta ZA, et al. Global, Regional, and National Cancer Incidence, Mortality, Years of Life Lost, Years Lived With Disability, and Disability-Adjusted Life-years for 32 Cancer Groups, 1990 to 2015: A Systematic Analysis for the Global Burden of Disease Study. JAMA oncology. 2016. 10.1001/jamaoncol.2016.5688. 27918777. 10.1001/jamaoncol.2016.5688 PMC6103527 27918777

[5] Sadjadi A, Nouraie M, Mohagheghi MA, Mousavi-Jarrahi A, Malekezadeh R, Parkin DM. Cancer occurrence in Iran in 2002, an international perspective. Asian Pacific journal of cancer prevention: APJCP. 2005;6(3):359–63. . 16236000

[6] Mousavi SM, Gouya MM, Ramazani R, Davanlou M, Hajsadeghi N, Seddighi Z. Cancer incidence and mortality in Iran. Annals of oncology: official journal of the European Society for Medical Oncology. 2009;20(3):556–63. doi: 10.1093/annonc/mdn642 . 19073863 10.1093/annonc/mdn642

[7] Harirchi I, Karbakhsh M, Kashefi A, Momtahen AJ. Breast cancer in Iran: results of a multi-center study. Asian Pacific journal of cancer prevention: APJCP. 2004;5(1):24–7. . 15075000

[8] Skibinski A, Kuperwasser C. The origin of breast tumor heterogeneity. Oncogene. 2015;34(42):5309–16. doi: 10.1038/onc.2014.475 ; PubMed Central PMCID: PMC4734640. 25703331 10.1038/onc.2014.475 PMC4734640

[9] Sana M, Malik HJ. Current and emerging breast cancer biomarkers. Journal of cancer research and therapeutics. 2015;11(3):508–13. doi: 10.4103/0973-1482.163698 . 26458575 10.4103/0973-1482.163698

[10] Levine AJ, Momand J, Finlay CA. The p53 tumour suppressor gene. Nature. 1991;351(6326):453–6. doi: 10.1038/351453a0 . 2046748 10.1038/351453a0

[11] Blaszyk H, Hartmann A, Cunningham JM, Schaid D, Wold LE, Kovach JS, et al A prospective trial of midwest breast cancer patients: a p53 gene mutation is the most important predictor of adverse outcome. International journal of cancer. 2000;89(1):32–8. . 10719728 10.1002/(sici)1097-0215(20000120)89:1<32::aid-ijc6>3.0.co;2-g

[12] Jung SY, Jeong J, Shin SH, Kwon Y, Kim EA, Ko KL, et al Accumulation of p53 determined by immunohistochemistry as a prognostic marker in node negative breast cancer; analysis according to St Gallen consensus and intrinsic subtypes. Journal of surgical oncology. 2011;103(3):207–11. doi: 10.1002/jso.21819 . 21337548 10.1002/jso.21819

[13] Silvestrini R, Benini E, Daidone MG, Veneroni S, Boracchi P, Cappelletti V, et al p53 as an independent prognostic marker in lymph node-negative breast cancer patients. Journal of the National Cancer Institute. 1993;85(12):965–70. . 8496982 10.1093/jnci/85.12.965

[14] Korkolis DP, Tsoli E, Fouskakis D, Yiotis J, Koullias GJ, Giannopoulos D, et al Tumor histology and stage but not p53, Her2-neu or cathepsin-D expression are independent prognostic factors in breast cancer patients. Anticancer research. 2004;24(3b):2061–8. . 15274401

[15] Elston CW, Ellis IO. Pathological prognostic factors in breast cancer. I. The value of histological grade in breast cancer: experience from a large study with long-term follow-up. Histopathology. 1991;19(5):403–10. . 1757079 10.1111/j.1365-2559.1991.tb00229.x

[16] Fine JP, Gray RJ. A Proportional Hazards Model for the Subdistribution of a Competing Risk. Journal of the American Statistical Association. 1999;94(446):496–509. doi: 10.1080/01621459.1999.10474144

[17] Gasparini G, Toi M, Verderio P, Ranieri G, Dante S, Bonoldi E, et al Prognostic significance of p53, angiogenesis, and other conventional features in operable breast cancer: subanalysis in node-positive and node-negative patients. International journal of oncology. 1998;12(5):1117–25. . 9538138 10.3892/ijo.12.5.1117

[18] Mirza AN, Mirza NQ, Vlastos G, Singletary SE. Prognostic factors in node-negative breast cancer: a review of studies with sample size more than 200 and follow-up more than 5 years. Annals of surgery. 2002;235(1):10–26. ; PubMed Central PMCID: PMC1422391. 11753038 10.1097/00000658-200201000-00003 PMC1422391

[19] Falette N, Paperin MP, Treilleux I, Gratadour AC, Peloux N, Mignotte H, et al Prognostic value of P53 gene mutations in a large series of node-negative breast cancer patients. Cancer research. 1998;58(7):1451–5. . 9537247

[20] Soontrapornchai P, Chanvitan A, Koontongkaew S, Sunpaweravong S. The prognostic value of p53 immunostaining in node-negative breast carcinoma. Journal of the Medical Association of Thailand = Chotmaihet thangphaet. 2007;90(9):1833–8. . 17957927

[21] Kroger N, Milde-Langosch K, Riethdorf S, Schmoor C, Schumacher M, Zander AR, et al Prognostic and predictive effects of immunohistochemical factors in high-risk primary breast cancer patients. Clinical cancer research: an official journal of the American Association for Cancer Research. 2006;12(1):159–68. doi: 10.1158/1078-0432.CCR-05-1340 . 16397038 10.1158/1078-0432.CCR-05-1340

[22] Vegran F, Rebucci M, Chevrier S, Cadouot M, Boidot R, Lizard-Nacol S. Only missense mutations affecting the DNA binding domain of p53 influence outcomes in patients with breast carcinoma. PloS one. 2013;8(1):e55103 doi: 10.1371/journal.pone.0055103 ; PubMed Central PMCID: PMC3554672. 23359294 10.1371/journal.pone.0055103 PMC3554672

[23] Sjogren S, Inganas M, Norberg T, Lindgren A, Nordgren H, Holmberg L, et al The p53 gene in breast cancer: prognostic value of complementary DNA sequencing versus immunohistochemistry. Journal of the National Cancer Institute. 1996;88(3–4):173–82. . 8632491 10.1093/jnci/88.3-4.173

[24] Mylonas I, Makovitzky J, Jeschke U, Briese V, Friese K, Gerber B. Expression of Her2/neu, steroid receptors (ER and PR), Ki67 and p53 in invasive mammary ductal carcinoma associated with ductal carcinoma In Situ (DCIS) Versus invasive breast cancer alone. Anticancer research. 2005;25(3A):1719–23. . 16033090

[25] Liu C, Zhang H, Shuang C, Lu Y, Jin F, Xu H, et al Alterations of ER, PR, HER-2/neu, and P53 protein expression in ductal breast carcinomas and clinical implications. Medical oncology. 2010;27(3):747–52. doi: 10.1007/s12032-009-9279-8 . 19657752 10.1007/s12032-009-9279-8

[26] Shokouh TZ, Ezatollah A, Barand P. Interrelationships Between Ki67, HER2/neu, p53, ER, and PR Status and Their Associations With Tumor Grade and Lymph Node Involvement in Breast Carcinoma Subtypes: Retrospective-Observational Analytical Study. Medicine. 2015;94(32):e1359 doi: 10.1097/MD.0000000000001359 ; PubMed Central PMCID: PMC4616694. 26266392 10.1097/MD.0000000000001359 PMC4616694

